# Laparoscopic Right Hemicolectomy With Gastrocolic Trunk Resection for Advanced Transverse Colon Cancer

**DOI:** 10.7759/cureus.67471

**Published:** 2024-08-22

**Authors:** Yusuke Asada, Hiroki Ochiai, Takahisa Yoshikawa, Takeo Fukagawa, Noriaki Kameyama

**Affiliations:** 1 Department of Surgery, Ogikubo Hospital, Tokyo, JPN; 2 Department of Surgery, Teikyo University School of Medicine, Tokyo, JPN

**Keywords:** gastrocolic trunk, extended resection, right hemicolectomy, laparoscopic surgery, colon cancer

## Abstract

Locally advanced right-sided colon cancer sometimes requires advanced procedures in addition to normal complete mesocolic excision. We describe laparoscopic right hemicolectomy with gastrocolic trunk (GCT) resection. A 48-year-old woman was diagnosed with right transverse colon cancer and severe lymph node metastasis. Bulky lymph nodes were in contact with the superior mesenteric vein (SMV) that invaded the root of the GCT. Curative laparoscopic right hemicolectomy with GCT resection was performed. GCT resection was performed using both cranial and caudal approaches. First, we ligated the distal side of the GCT from the cranial side and dissected the mesocolonic root from the pancreas. Then, we moved to the caudal view. The root of the GCT was ligated, and the resected GCT was mobilized from the pancreatic head while carefully coagulating the anterior superior pancreaticoduodenal veins (ASPDVs) using an ultrasonically activated device (USAD). The patient’s postoperative course was favorable. Approaching the GCT from both the cranial and caudal sides, considering the limited handling axis of laparoscopy, is useful for performing this procedure safely. The cranial approach is important for creating a cranial safety zone before transitioning to the caudal approach. The pitfall is that the ASPDVs should not be managed in this step because the head of the USAD will contact the pancreatic head owing to the handling axis. ASPDVs should be managed using the caudal approach with a cranial safety zone. Although rarely performed, this procedure is sometimes essential for the treatment of advanced right-sided colon cancer.

## Introduction

Complete mesocolic excision with central vascular ligation for colon cancer, which usually involves lymph node dissection along the superior mesenteric vein (SMV) in the right colon, is now a standard and basic procedure, even in laparoscopic surgery [1-4]. However, tumor progression near the pancreas, duodenum, and SMV sometimes requires extremely invasive and advanced procedures for en bloc resection, e.g., pancreaticoduodenectomy and/or SMV resection, usually performed in open surgery [5-8]. Here, we describe laparoscopic right hemicolectomy with gastrocolic trunk (GCT) resection and preservation of other organs for advanced transverse colon cancer with severe lymph node metastasis. This procedure was necessary and sufficient for this patient, and to our best knowledge, this is the first report to describe such a procedure in detail.

## Case presentation

**Figure 1:**
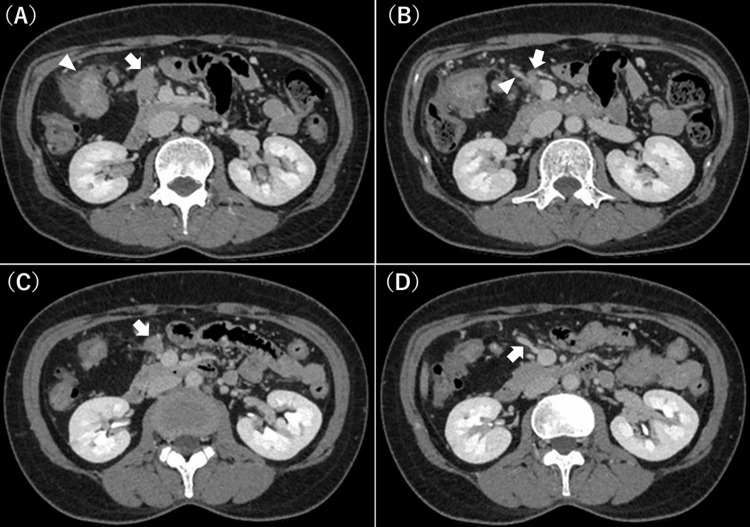
Computed tomography findings at the first visit (A, B) and after preoperative chemotherapy (C, D) (A) At the first visit, bulky regional lymph nodes (arrow) are in contact with the pancreatic head. The primary tumor is shown in the right transverse colon (arrowhead). As shown, the primary tumor is circumferential, and the colonoscope could not pass through the lesion. (B) Lymph nodes (arrow) are also in contact with the superior mesenteric vein (SMV) invading the root of the gastrocolic trunk (GCT) (arrowhead). (C) After chemotherapy, a thin fat layer appears between the shrunken lymph nodes (arrow) and the pancreatic head or SMV. (D) The root of the GCT (arrow), which should be sacrificed for en bloc lymph node dissection, is able to be encircled

A 48-year-old woman presented with anemia. A colonoscopy revealed circumferential advanced cancer in the right transverse colon, and a biopsy revealed adenocarcinoma and wild-type RAS. Her hemoglobin level was 8.5 g/dL, and her carcinoembryonic antigen (CEA) level was 20.9 ng/mL. Computed tomography showed severe lymph node metastasis. Bulky regional lymph nodes were in contact with the pancreatic head (Figure 1A). Additionally, lymph nodes were in contact with the SMV invading the root of the GCT (Figure 1B), and a single enlarged subpyloric lymph node was observed. The diagnosis was T4aN2aM1a (extra-regional lymph node) stage IVA, according to the 8th Union for International Cancer Control classification. The situation was considered technically and oncologically challenging for immediate resection; therefore, we decided to initiate chemotherapy first, following the guidelines for unresectable colorectal cancer. Furthermore, because the primary tumor was circumferential and the colonoscope could not pass through the lesion, we decided to construct a stoma before starting chemotherapy. After loop ileostomy construction, we performed six cycles of 5-fluorouracil, leucovorin, and oxaliplatin (mFOLFOX6) plus panitumumab therapy. After chemotherapy, each lesion shrank, and a thin fat layer appeared between the lymph nodes and the pancreatic head or SMV (Figure 1C). The root of the GCT, which should be sacrificed for en bloc lymph node dissection, was able to be encircled (Figure 1D). All lesions became technically resectable. Furthermore, the CEA level normalized from 20.9 to 3.1 ng/mL, indicating systemic disease control. Consequently, we decided to proceed with curative surgery at this time.

**Figure 2:**
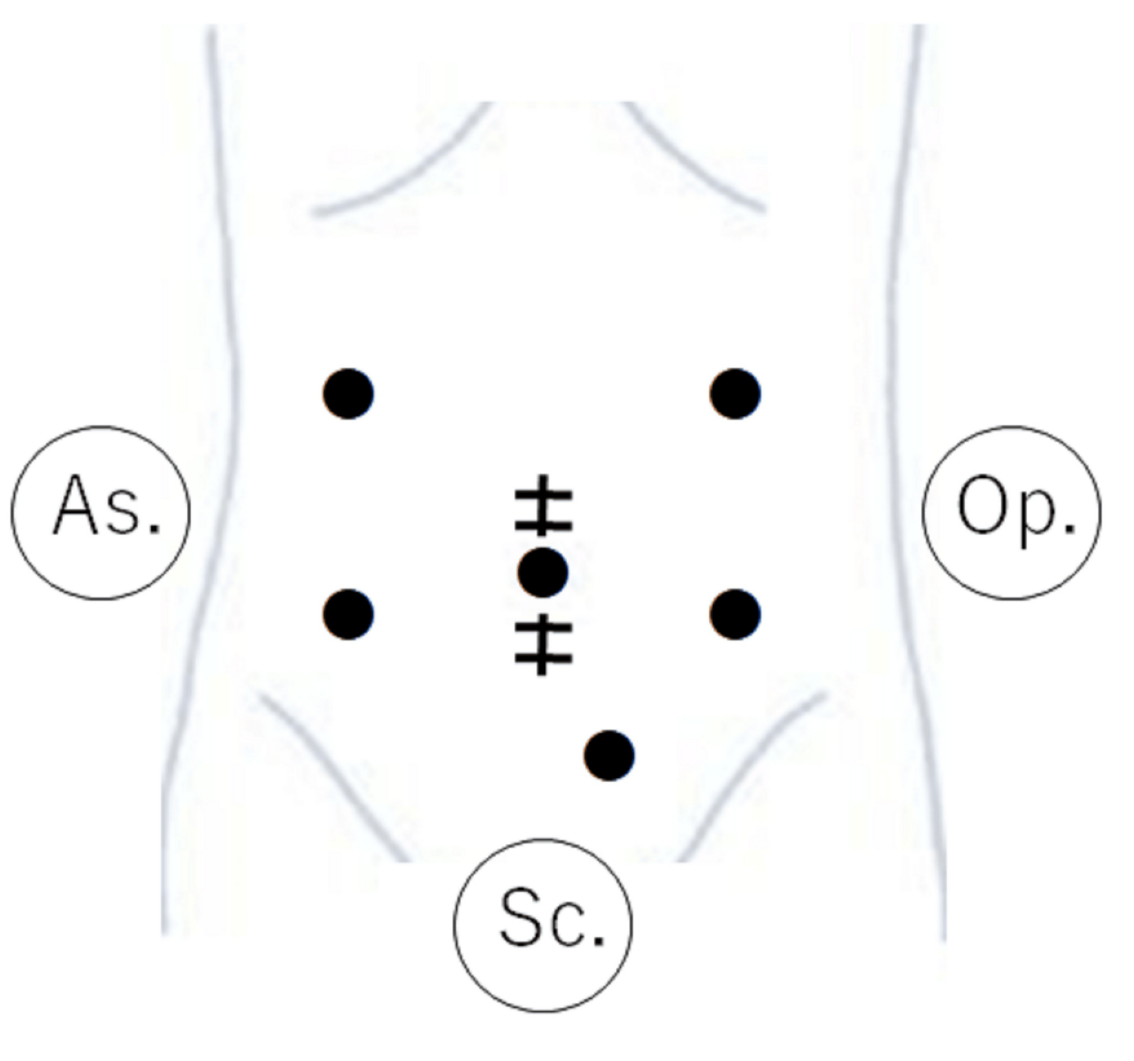
Setup of the surgery (six ports) In small laparotomy for extracorporeal anastomosis, the first port at the umbilical site is for the scopist (Sc.). Additional five ports: left upper/lower and suprapubic ports for the operator (Op.), and right upper/lower ports for the assistant (As.). This figure is our original

**Figure 3:**
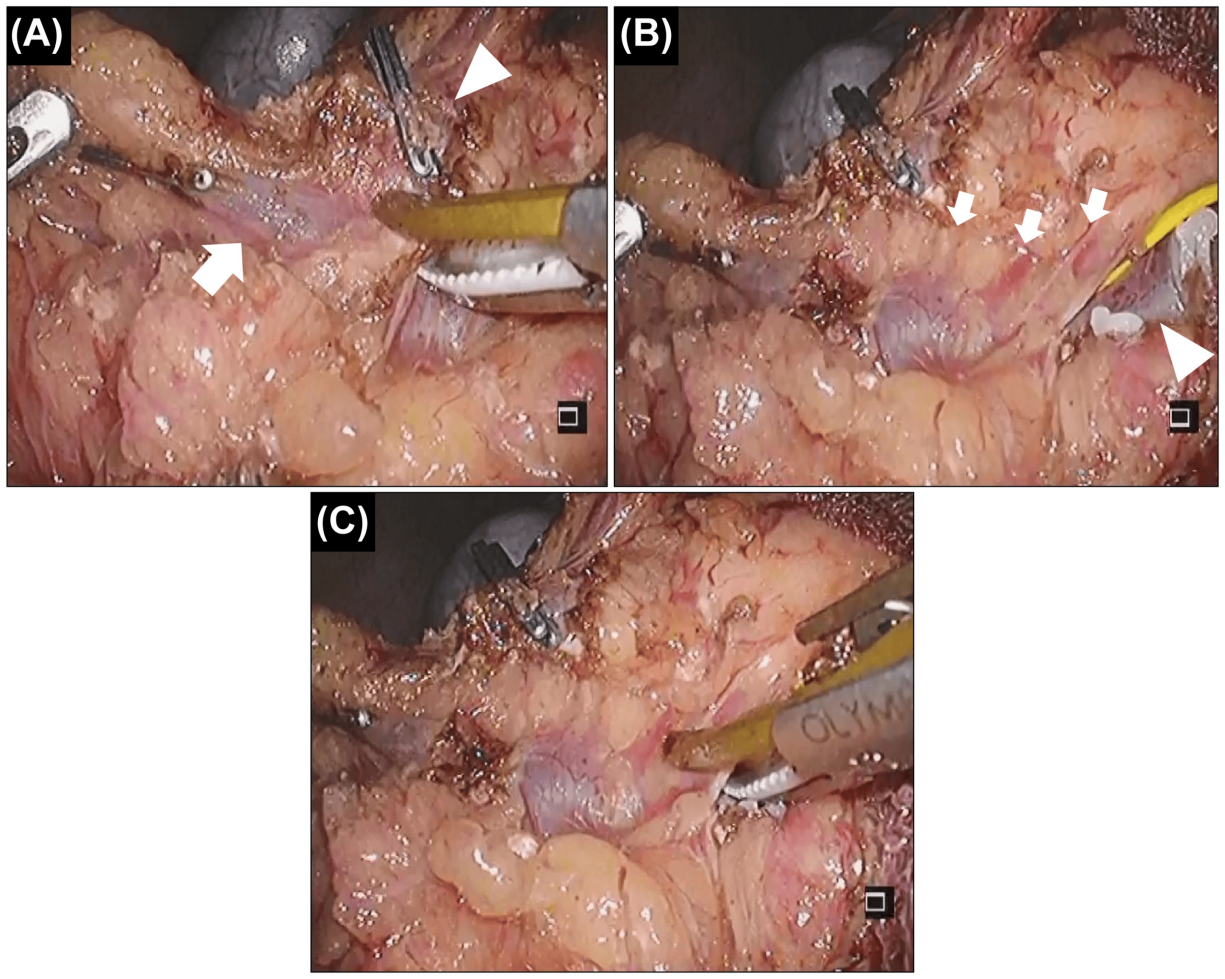
The gastrocolic trunk (GCT) is approached from the cranial side (A) Dissection of the resected GCT (arrow: the distal side (i.e., the right gastroepiploic vein) was already ligated) from the gastroepiploic artery (arrowhead: already ligated for subpyloric lymph node dissection in this patient). (B) Anterior superior pancreaticoduodenal veins (ASPDVs) in the cranial view (arrow). The root of the GCT is shown (arrowhead). (C) The head of the ultrasonically activated device will contact the pancreatic head if we manage the ASPDVs from the cranial side. Thus, this approach is dangerous

**Figure 4:**
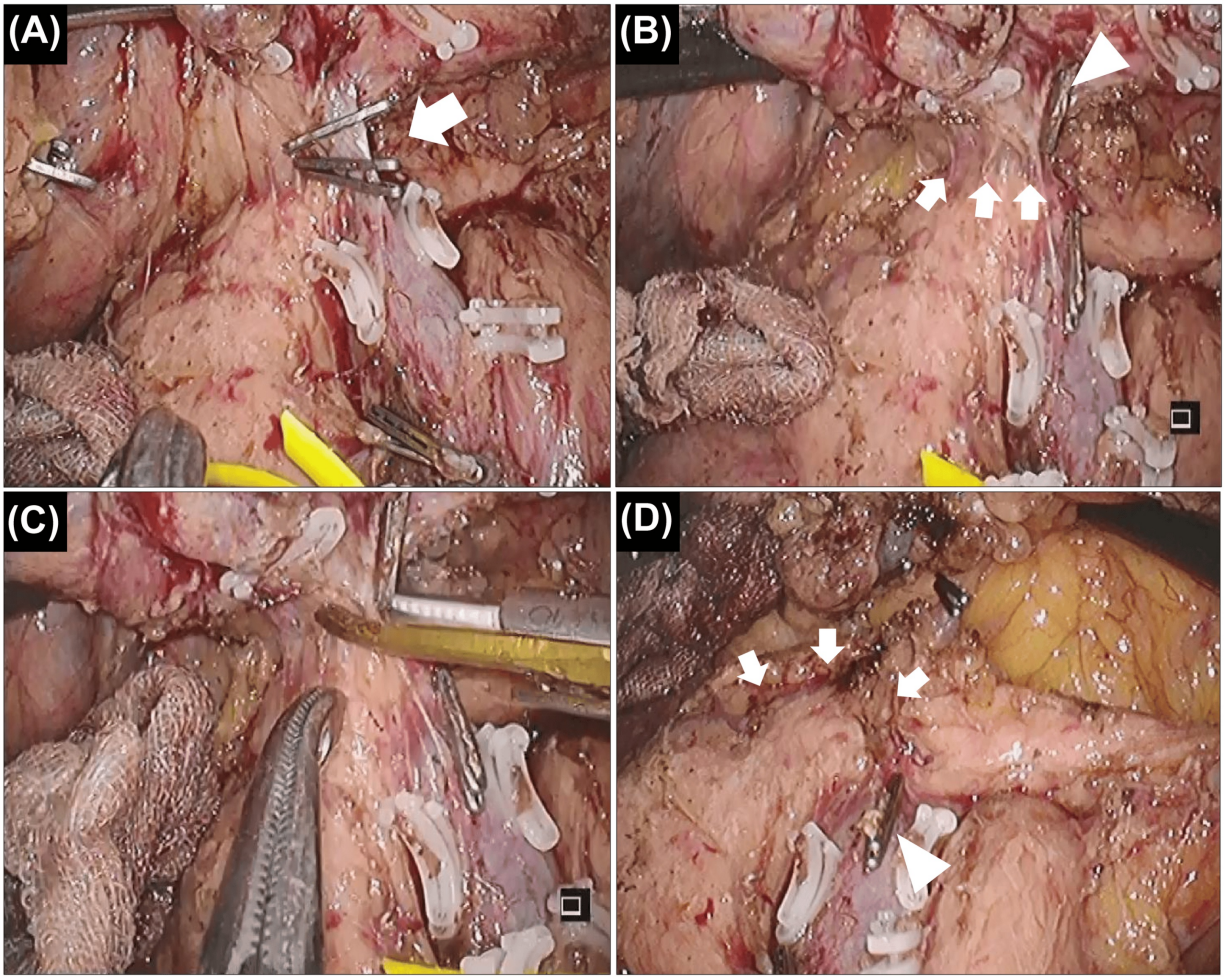
The gastrocolic trunk (GCT) is approached from the caudal side (A) Ligation of the root of the GCT (arrow). (B) Anterior superior pancreaticoduodenal veins (ASPDVs) in the caudal view (arrow). The GCT is resected (arrowhead). (C) Managing the ASPDVs is safe using this approach because the head of the ultrasonically activated device will make contact with the cranial safety zone instead of the pancreatic head. (D) En bloc resection is achieved. The stump of the ASPDVs (arrow) and the root of the GCT (arrowhead) are shown

Laparoscopic right hemicolectomy with GCT resection and subpyloric lymph node dissection was performed. The surgical setup is shown in Figure 2. The operator used the left lower port (dominant right hand) and the suprapubic port. First, we flipped the entire small bowel and approached the mesenteric root. The mesocolon was mobilized from the retroperitoneum. Once the root of the small bowel mesentery was expanded, it became very easy to safely mobilize the right hemicolon from the retroperitoneum, including the duodenum and pancreatic head. Subsequently, the ileocolic, right colic, and middle colic vessels were ligated. Although the root of the GCT could also be encircled in this step in this patient, we did not ligate it to prevent blood congestion. GCT resection was performed using both cranial and caudal approaches. First, we ligated the distal side of the GCT (i.e., the right gastroepiploic vein) from the cranial side and dissected the root of the mesocolon from the pancreas (Figure 3A-3C). In this step, the operator used the left upper port for the dominant right hand. Subsequently, we returned to the caudal view. The root of the GCT was ligated, and the resected GCT was mobilized from the pancreatic head while carefully coagulating the anterior superior pancreaticoduodenal veins (ASPDVs) using an ultrasonically activated device (USAD) (Figure 4A-4D).

Subpyloric lymph node dissection was performed independently. The operative time and blood loss volume were 451 minutes and 75 mL, respectively. The patient’s postoperative course was uneventful, and the pathological diagnosis was T3N1bM1a, stage IVA. Regional lymph node metastasis was identified along the middle colic vessels, including the root near the SMV (three out of 35 harvested lymph nodes), and a single subpyloric lymph node metastasis was observed among the nine harvested lymph nodes, consistent with the preoperative diagnosis. The chemotherapeutic effect was classified as Grade 1a according to the Japanese Classification of Colorectal, Appendiceal, and Anal Carcinoma (Ninth Edition). We administered six additional cycles of mFOLFOX6 as adjuvant chemotherapy. The patient was recurrence-free 12 months after curative surgery.

## Discussion

Here, we described laparoscopic right hemicolectomy with GCT resection for advanced transverse colon cancer with severe lymph node metastasis. Ligation of the root of the GCT itself is not too difficult, which is currently the routine step of laparoscopic pancreaticoduodenectomy [9,10]. Few case reports have described this procedure for colon cancer [11,12]. However, to resect the GCT while preserving the pancreas, the GCT must be mobilized from the pancreatic head while managing the ASPDVs between them. This procedure is rarely performed, and severe bleeding and pancreatic fistulae should be considered.

Approaching the GCT from both the cranial and caudal sides, considering the limited handling axis of laparoscopy, is useful for performing this procedure safely. Furthermore, regardless of the surgical approach (open, laparoscopic, or robotic), we believe that approaching critical points from multiple directions is a fundamental principle for ensuring surgical safety. This is an application of the “pincer approach,” a safe laparoscopic approach to the root of transverse mesocolon demonstrated by Egi et al. [13]. First, we approached the GCT from the cranial side. We ligated the distal side of the GCT (i.e., the right gastroepiploic vein) and dissected the root of the mesocolon from the pancreas. This step is important for creating a cranial safety zone before transitioning to the caudal approach. Although dissection of the resected GCT from the gastroepiploic artery is needed (Figure 3A), management of the ASPDVs should not be performed in this step because the head of the USAD will contact the pancreatic head owing to the handling axis (Figure 3C). ASPDVs should be managed using the caudal approach in laparoscopic surgery, although robotic surgery with a free-handling axis may overcome this problem. We then approached the GCT caudally. In this approach, managing the ASPDVs, which remain a screen between the mobilizing GCT and the pancreatic head, is safe because the head of the USAD is directed to the cranial safety zone (Figure 4C). There are numerous tiny ASPDVs, and the USAD is the optimal device to manage these tiny vessels.

## Conclusions

We described laparoscopic right hemicolectomy with GCT resection in detail. Although this procedure is rarely performed, it is sometimes essential for the treatment of advanced right-sided colon cancer. Approaching the GCT from both the cranial and caudal sides, considering the limited handling axis of laparoscopy, may be the key to safely performing this procedure.
